# Tuberculosis in Ukrainian War Refugees and Migrants in the Czech Republic and Slovakia: A Molecular Epidemiological Study

**DOI:** 10.1007/s44197-023-00166-5

**Published:** 2023-12-04

**Authors:** Matúš Dohál, Věra Dvořáková, Miluše Šperková, Martina Pinková, Arash Ghodousi, Maryam Omrani, Igor Porvazník, Erik Michael Rasmussen, Mária Škereňová, Michaela Krivošová, Jiří Wallenfels, Olha Konstantynovska, Timothy M. Walker, Vladyslav Nikolayevskyy, Daniela Maria Cirillo, Ivan Solovič, Juraj Mokrý

**Affiliations:** 1 Comenius University Bratislava, Malá Hora 4A, 036 01 Martin, Slovak Republic; 2 National Institute of Public Health, Prague, Czech Republic; 3 IRCCS San Raffaele Scientific Institute, Milan, Italy; 4 San Raffaele University, Milan, Italy; 5 University of Milan, Milan, Italy; 6 National Institute of Tuberculosis, Lung Diseases and Thoracic Surgery, Vyšné Hágy, Slovak Republic; 7 Catholic University, Ružomberok, Slovak Republic; 8 Statens Serum Institut, Copenhagen, Denmark; 9 University Hospital Bulovka, Prague, Czech Republic; 10 V.N. Karazin Kharkiv National University, Kharkiv, Ukraine; 11 Oxford University Clinical Research Unit, Ho Chi Minh City, Vietnam; 12 University of Oxford, Oxford, UK; 13 Imperial College London, London, UK

**Keywords:** Tuberculosis epidemiology, Migration, Control of tuberculosis, Refugees

## Abstract

**Background:**

The war in Ukraine has led to significant migration to neighboring countries, raising public health concerns. Notable tuberculosis (TB) incidence rates in Ukraine emphasize the immediate requirement to prioritize approaches that interrupt the spread and prevent new infections.

**Methods:**

We conducted a prospective genomic surveillance study to assess migration's impact on TB epidemiology in the Czech Republic and Slovakia. *Mycobacterium tuberculosis* isolates from Ukrainian war refugees and migrants, collected from September 2021 to December 2022 were analyzed alongside 1574 isolates obtained from Ukraine, the Czech Republic, and Slovakia.

**Results:**

Our study revealed alarming results, with historically the highest number of Ukrainian tuberculosis patients detected in the host countries. The increasing number of cases of multidrug-resistant TB, significantly linked with Beijing lineage 2.2.1 (*p* < 0.0001), also presents substantial obstacles to control endeavors. The genomic analysis identified the three highly related genomic clusters, indicating the recent TB transmission among migrant populations. The largest clusters comprised war refugees diagnosed in the Czech Republic, TB patients from various regions of Ukraine, and incarcerated individuals diagnosed with pulmonary TB specialized facility in the Kharkiv region, Ukraine, pointing to a national transmission sequence that has persisted for over 14 years.

**Conclusions:**

The data showed that most infections were likely the result of reactivation of latent disease or exposure to TB before migration rather than recent transmission occurring within the host country. However, close monitoring, appropriate treatment, careful surveillance, and social support are crucial in mitigating future risks, though there is currently no evidence of local transmission in EU countries.

**Supplementary Information:**

The online version contains supplementary material available at 10.1007/s44197-023-00166-5.

## Introduction

In February 2022, armed conflict in Ukraine led to mass migration of nationals to neighboring EU countries. Over 8 million refugees from Ukraine were recorded in Europe as of February 7, 2023, the largest since World War II [1, 2]. Poland, Czech Republic, Romania, and Slovakia faced the highest refugee influx. The ongoing conflict has put refugees at increased risk of tuberculosis due to poor living conditions and limited healthcare access, impacting both physical and emotional well-being [3, 4].

According to the World Health Organization (WHO), Ukraine has one of Europe’s highest TB incidence rates, and the ongoing conflict has further exacerbated the situation. In 2021, Ukraine had an estimated incidence of 73 per 100,000, compared to 9.5 per 100,000 across European Union and European Economic Area. If we hypothetically consider the presence of eight million Ukrainian refugees dispersed throughout Europe, it can be postulated that around 23% of them have been infected with latent TB based on the global prevalence established by the tuberculin skin test (TST) and interferon-gamma release assay (IGRA) [5]. Considering statistical data, we can infer that a percentage ranging from 5 to 15% of those with latent TB will eventually develop active disease at some point in their lives [6]. Approximately 21% of cases were diagnosed with rifampicin-/multidrug-resistant TB (RR/MDR-TB), and Ukraine is among the top 30 countries with the highest incidence of drug-resistant cases [7–9]. Globally, only around a third of RR/MDR-TB cases are detected and receive appropriate treatment [10]. As a result, the previously stable situation of DR-TB is likely to encounter significant shifts, especially in countries with low TB incidence that are facing a substantial influx of war refugees. Despite this, Ukraine is not considered a high-TB incidence country, and neither European Centre for Disease Prevention and Control (ECDC) nor WHO guidelines recommend universal testing of migrants and refugees for TB infection [11].

In the context of TB in refugees and migrants, whole genome sequencing (WGS) can help elucidate the disease's transmission dynamics and provide detailed insights into phylogeny and resistance [12, 13]. Providing a high-resolution view of TB strains' genetic diversity can help guide public health interventions and control strategies to reduce the global burden of this lethal disease.

We performed a WGS-based epidemiological analysis on all *Mycobacterium tuberculosis complex* (*MTBC*) isolates obtained from Ukrainian war refugees and migrants who arrived in the Czech Republic and Slovakia between September 1, 2021, and December 31, 2022. We attempted to characterize whether the genetic relatedness of the studied strains corresponds to recent transmission in the host country or to independent transmission events in Ukraine before migration by comparing the isolates with 1574 *MTBC* isolates originally from Ukraine, the Czech Republic, and Slovakia.

## Materials and Methods

### Ukrainian Refugees and Migrants

The study population included all Ukrainian citizens who were diagnosed with TB in Slovakia and the Czech Republic from 1 September 2021 to 31 December 2022. The study start was determined by the increasing migration rate from September 2021, which resulted from the escalating war tensions between Russia and Ukraine while also coinciding with the removal of COVID-19 restrictions in the host countries. All isolates had patient data available on the date of first TB diagnosis, age, sex, and date of arrival in the country. Based on this information, the patients were classified into two distinct groups: migrants and war refugees.

Phenotypic drug susceptibility testing (pDST) was performed on Lowenstein–Jensen (LJ) medium for all drugs; for pyrazinamide (PZA), we used the BACTEC MGIT 960 system. The media contained drugs at critical concentrations (CC) recommended by the WHO [14].

DNA extraction and WGS were performed according to the procedure published in our previous study [15].

### International TB Transmission Investigation

MTBC isolates from Slovakia, Czech Republic, and Ukraine were analyzed to study international transmission chains and infection origin. Brief country overviews and aggregate data are below:

#### Czech Republic

All accessible *MTBC* sequences (*n* = 143) from the Czech Republic were employed in this analysis. MDR isolates (*n* = 65) were collected during routine surveillance from January 2005 to December 2020 and have been described previously (accession number PRJEB48710) [15]. All mono- and polyresistant strains (*n* = 78) obtained during health surveillance from January 2017 to December 2020 were analyzed. DNA extraction and WGS were performed as mentioned above (see section Ukrainian refugees and migrants).

#### Slovakia

All accessible *MTBC* sequences (*n* = 61) from Slovakia were employed in this analysis, including all MDR, mono- and polyresistant *MTBC* isolated at National Reference Laboratory for Mycobacteria between January 2017 and December 2020 and drug-susceptible isolates collected between June 2019 and March 2020. DNA extraction and WGS were performed as mentioned above (see section Ukrainian refugees and migrants). Twelve MDR and pre-XDR isolates have been described previously (accession number PRJEB43174) [16].

#### Ukraine

The study incorporated a cohort of 1370 *MTBC* isolates sourced from the state of Ukraine.

A total of 1184 isolates collected between November 2009 and May 2014 were obtained from the Central Reference Laboratory on Tuberculosis Microbiological Diagnostics, Ministry of Health, Kyiv, Ukraine. In total, 32.6% of the isolates were found to be RR, with 9.2% of these exhibiting MDR. These isolates were selected based on population-based surveys carried out in hospitals and clinics across diverse regions in Ukraine to assess the prevalence of anti-tuberculosis drug resistance among *MTBC* isolates, and sequences were described previously (accession number SRP128089) [17, 18].

Furthermore, a set of 186 isolates, previously uncharacterised, was obtained from V.N. Karazin Kharkiv National University, Kharkiv, Ukraine. These strains were isolated from prison inmates diagnosed with pulmonary TB treated at a specialized facility in Kharkiv region, Ukraine.

For more information on DNA isolation and WGS, see Supplementary Data 1.1 and 1.2.

### Statistics

The association between lineages and resistance profile was assessed using Fisher’s exact test (two-sided) using GraphPad Prism version 8.0.1.

### Bioinformatic Analysis

The sequenced reads were mapped to *MTBC* H37Rv ATCC 27294 as reference genomes using the MTBseq pipeline by application of the mem algorithm of the Burrows–Wheeler alignment tool. Duplicated reads were marked using the Picard tool (https://github.com/broadinstitute/picard), and local realignment of reads around insertions/deletions was performed using the Genome Analysis Toolkit. High-quality SNPs were called with Samtools mpileup using the following thresholds: minimum mapping quality of 20, minimum base quality at a position of 20, minimum read depth at a position of 8×, and maximum strand bias for a position of 90%. Samples not meeting these criteria were excluded from further analysis (*n* = 158).

In order to detect the resistant subpopulations or heteroresistance, the variant calling was performed using the minimum mapping quality of 20, minimum base quality at a position of 20, minimum read depth at a position of 2×, and maximum strand bias for a position of 10%. Moreover, an *in-house* script was used in order to detect large deletions in drug resistance-associated genes.

A maximum-likelihood phylogenetic tree was constructed from 12,468 reliable SNPs within the dataset of 1368 samples with respect to the reference genome with RAxML-NG v. 1.0.2, employing the '–model GTR + G + ASC_LEWIS' option. The general time reversible (GTR) model of nucleotide substitution with the gamma model of rate heterogeneity was utilized, and 100 independent runs were performed using distinct starting trees. Support values were obtained through 100 rounds of "Rapid" bootstrapping, and the best-scoring maximum-likelihood topology was "midpoint rooted" using FigTree (https://github.com/cdeanj/figtree). The resulting topology was annotated and visually enhanced using the iTOL online tool (https://itol.embl.de).

## Results

### Sample Collection and Analysis of Epidemiological Trends of TB Among Ukrainian Refugees and Migrants

**Fig. 1:**
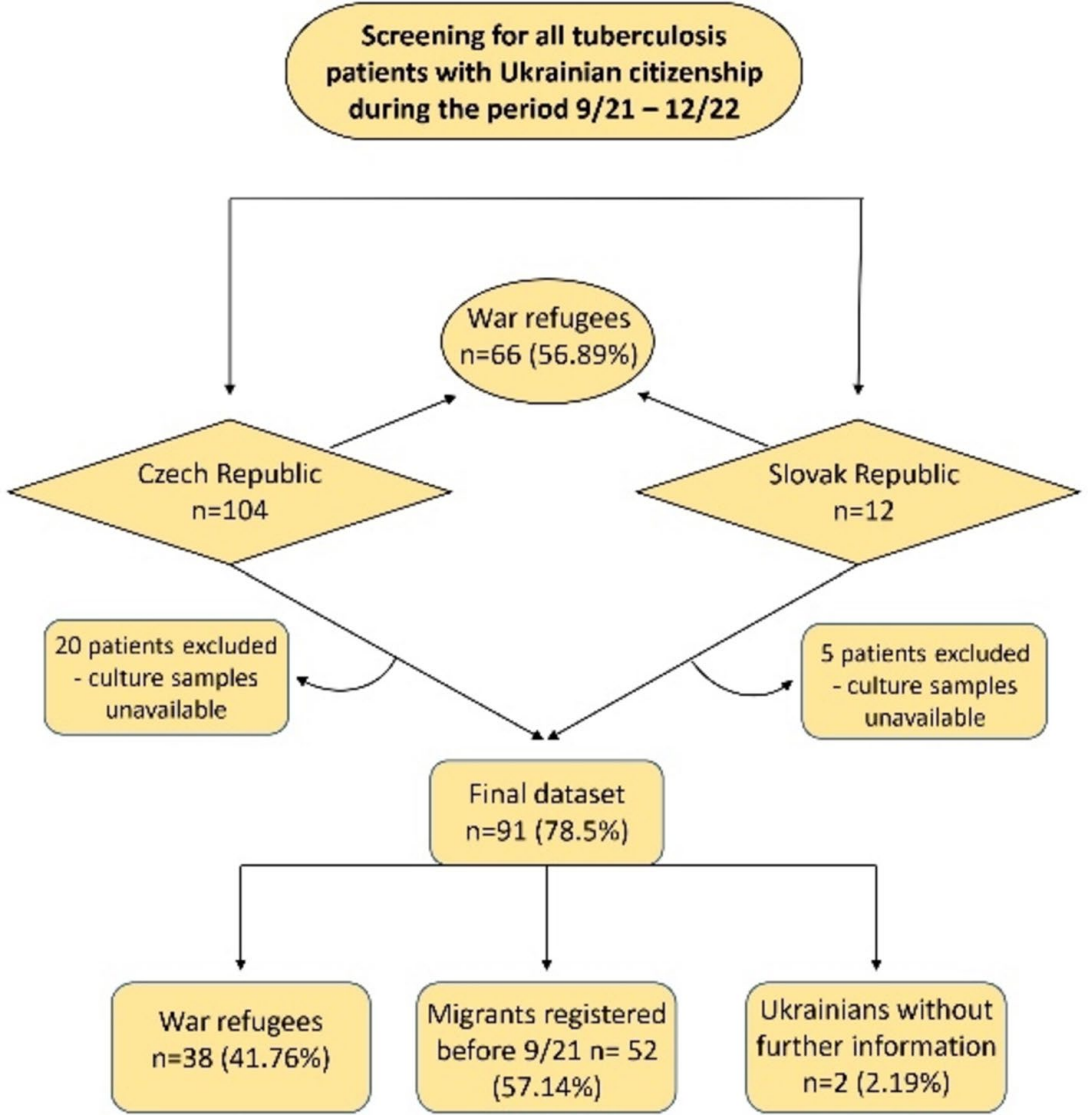
Flow chart showing the number of isolates from Ukrainian refugees and migrants included in epidemiological investigations into TB cases from September 1, 2021, to December 31, 2022, in the Czech Republic and Slovakia

From September 1, 2021, to December 31, 2022, a cumulative count of 116 TB cases was reported among Ukrainian refugees and migrants in the Czech Republic (*n* = 104) and Slovakia (*n* = 12). Culture-confirmed isolates were obtained from 91 patients (91/116; 78.45%) who underwent WGS (Fig. 1, Table 1, Supplementary Fig. S1).

**Table 1:** Epidemiological data for 91 Ukrainian war refugees/migrants underwent whole genome sequencing

Epidemiological data	
*N* (%)	
*Age group*	
0–9	1 (1.1)
10–19	3 (3.3)
20–29	8 (8.8)
30–39	26 (28.6)
40–49	23 (25.3)
50–59	19 (20.9)
60–69	6 (6.6)
70–79	4 (4.4)
80–89	1 (1.1)
*Sex*	
Male	58 (63.7)
Female	33 (36.3)
*Diagnosis*	
Pulmonary TB	86 (94.51%)
Extrapulmonary TB	5 (5.49%)
*History of TB*	
Yes	1 (1.1)
No	90 (98.9)

*TB* tuberculosis

**Fig. 2:**
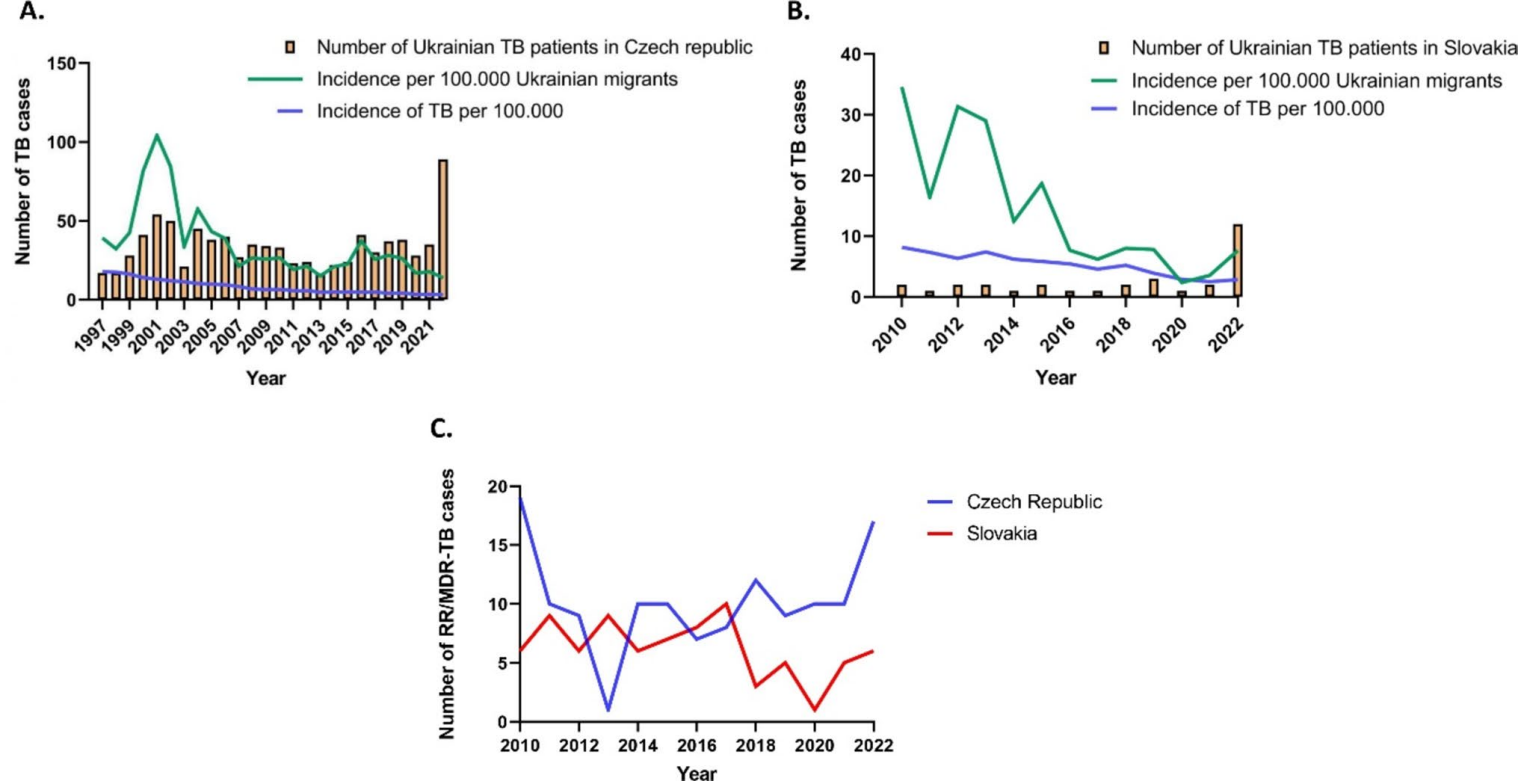
Trends in the incidence of TB among Ukrainian migrants in the Czech Republic from 1997 to 2022 (**A**) and Slovakia from 2010 to 2022 (**B**). Panel C shows the number of patients with RR/MDR-TB in the Czech Republic and Slovakia from 2010 to 2022. *data on the nationality of TB patients in Slovakia prior to 2010 was unavailable

Figure 2a, b illustrates the trends in TB incidence per 100,000 individuals among Ukrainian migrants in the Czech Republic and Slovakia over a span of 26 years and 13 years, respectively. Furthermore, it presents the annual number of diagnosed TB cases in the Czech Republic from 1997 to 2022 (Fig. 2a) and Slovakia from 2010 to 2022 (Fig. 2b). Despite the significant increase in the number of patients in 2022, there was a decrease in TB incidence (Fig. 2a). This trend is attributed to the arrival of 636,282 war refugees to the country due to the ongoing conflict. Among the total population of 156,881 Ukrainian war refugees in Slovakia, 12 TB cases were reported in 2022.

Furthermore, according to pDST, we observed the highest number of patients with RR and MDR-TB in the Czech Republic in the past 11 years (*n* = 17), out of which 8 (47.06%) were identified among war refugees (Fig. 2c). A total of 6 cases of MDR-TB were recorded in Slovakia in 2022, representing the highest number in the last 5 years (Fig. 2c). Of these, 3 cases (50%) were diagnosed in Ukrainian war refugees.

### Genotypic Resistance

Of the total 91 *MTBC* isolates from Ukrainian war refugees and migrants analyzed, 71.43% (65/91) were found to be drug-sensitive, 12.09% (11/91) exhibited resistance to at least one drug, and 16.48% (15/91) were classified as either MDR or pre-XDR.

We studied the sublineage-specific resistance profile and found that Beijing sublineage 2.2.1 was significantly associated with the RR/MDR (*p* < 0.0001; OR: 8.485; 95% CI 2.898–23.14) (Supplementary Fig. S2).

### Genomic Diversity of MTBC Isolates from Ukrainian War Refugees and Migrants in the Czech Republic and Slovakia

**Fig. 3:**
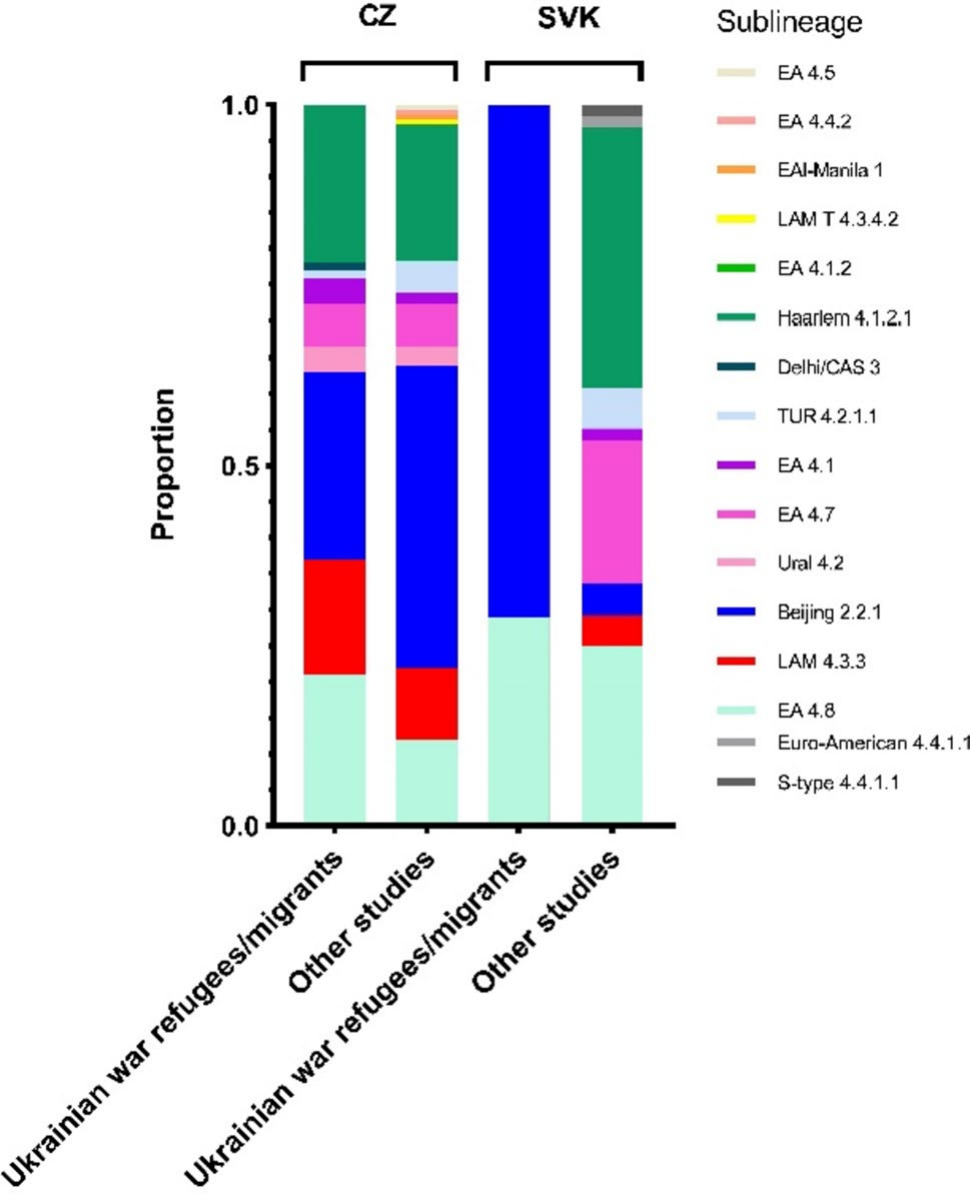
Variations in the genomic diversity of *Mtb* isolates collected during the present study and in previous studies conducted in the Czech Republic and Slovakia between 2005 and 2020. CZ—Czech Republic, SVK—Slovakia

The *MTBC* isolates were classified into three distinct lineages: lineage 4 (Euro-American, EA; 66/91), lineage 2 (Beijing; 24/91), and lineage 3 (Delhi/Central Asian, Delhi/CAS; 1/91). The strains were primarily categorized into nine sub-lineages: 4.1.2/Haarlem (21.98%%; 20/91), 4.8 EA (20.88%; 19/91), 4.3.3/LAM (16.48%; 15/91), 4.7/EA (5.49%; 5/91), 4.2/Ural (3.30%; 3/91), 4.1/EA (3.30%; 3/91), 4.2.1.1/TUR (1.10%; 1/91), 2.2.1/Beijing (26.37%; 24/91; of which W148 European/Russian clade 4/24; 16.67%) and 3.1.2.1/Delhi CAS (1.10%; 1/91). The prevalence of strains representing these sublineages remained constant and did not change significantly. On the contrary, in Slovakia, we noticed a significant change in the distribution of *MTBC* lineages (Fig. 3).

**Fig. 4:**
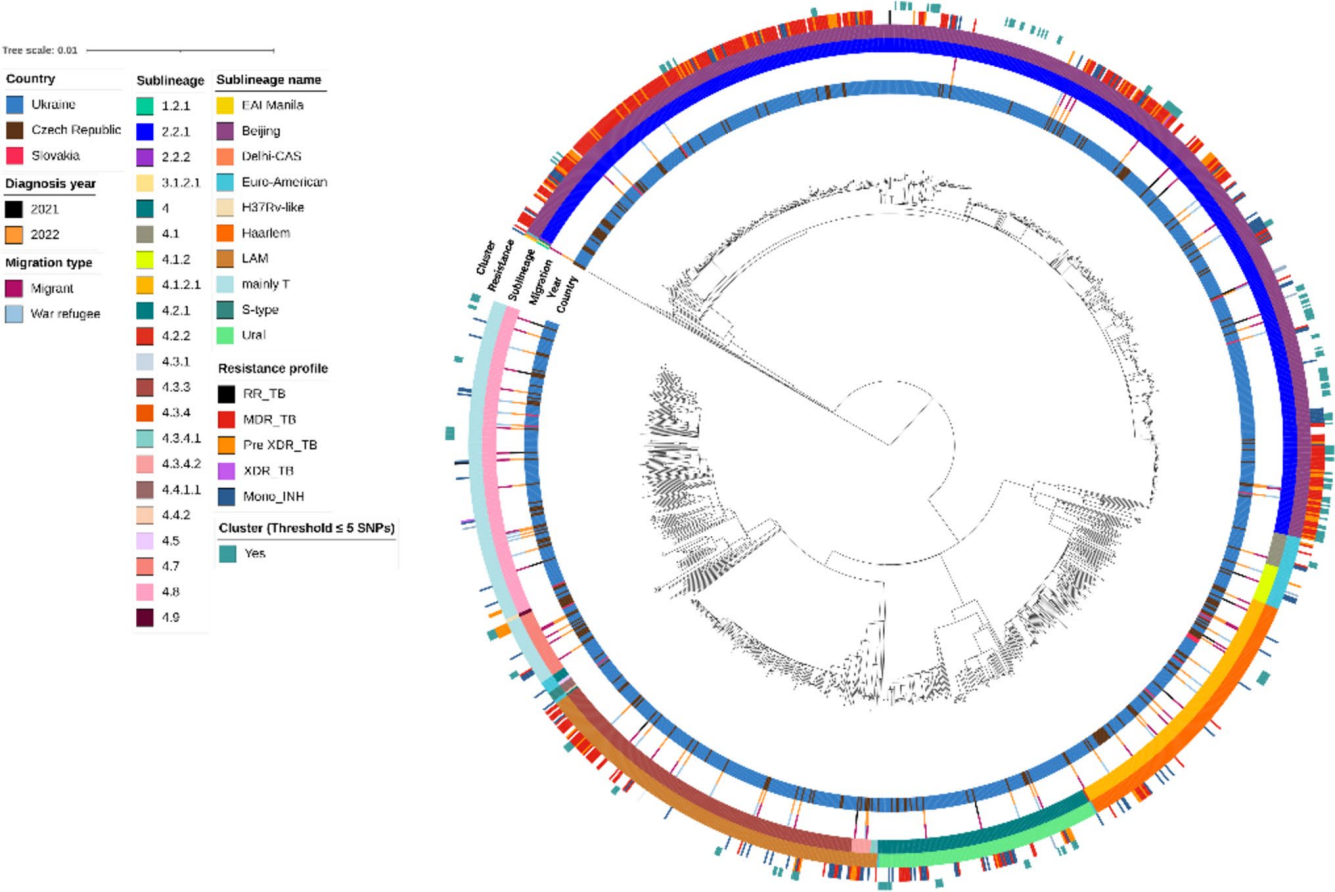
A maximum-likelihood phylogenetic tree was constructed from 12,468 reliable SNPs within the 1368 samples from the Czech Republic, Slovakia and Ukraine datasets. From the inner to the outer circles: country of TB diagnostics; year of TB diagnostics; migration status; sublineage; whole-genome sequencing-based drug-resistance profile; genomic cluster with a 5 SNP threshold

A phylogenetic tree constructed using the 1368 samples from the Czech Republic, Slovakia, and Ukraine was built to investigate the *MTBC* population structure and revealed the expected clustering by lineage with the prevalence of Beijing lineage (Fig. 4).

### MTBC Genomic Clusters/Transmission Analysis of Ukrainian War Refugees/Migrants

The pairwise SNP differences across the 91 isolates ranged from 0 to 652 (Supplementary Fig. S3). Among 91 *MTBC* isolates from Ukrainian war refugees and migrants, only 6 (8.79%) were grouped in three clusters, with a maximum number of isolates in each cluster being 2. There were 85 (85/91; 91.21%) genotypically unique isolates. One cluster comprising 2 patients was defined as a cross-border between the Czech Republic and Slovakia (0 SNPs difference; Supplementary Fig. S3). These 2 patients were infected in Ukraine, and while migrating after March 1, 2022, one patient stayed in Slovakia and one patient in the Czech Republic. Another cluster grouped 2 patients, one of whom has been registered as an asylum seeker in the Czech Republic since 2004, and the other came as a war refugee in 2022. Due to the relatively prolonged incubation period of TB, it can be presumed that these patients had previous direct contact or contact with other patients belonging to this cluster, whose sequencing data is unavailable. This data confirmed that there is a lack of clustering among strains from recent migrants/refugees, indicating their lack of genetic relatedness and, therefore, implying that transmission is unlikely to have occurred in the Czech Republic and Slovakia.

### International Transmission of TB Between War Refugees/Migrants and the General Population in the Czech Republic, Slovakia, and Ukraine

**Fig. 5:**
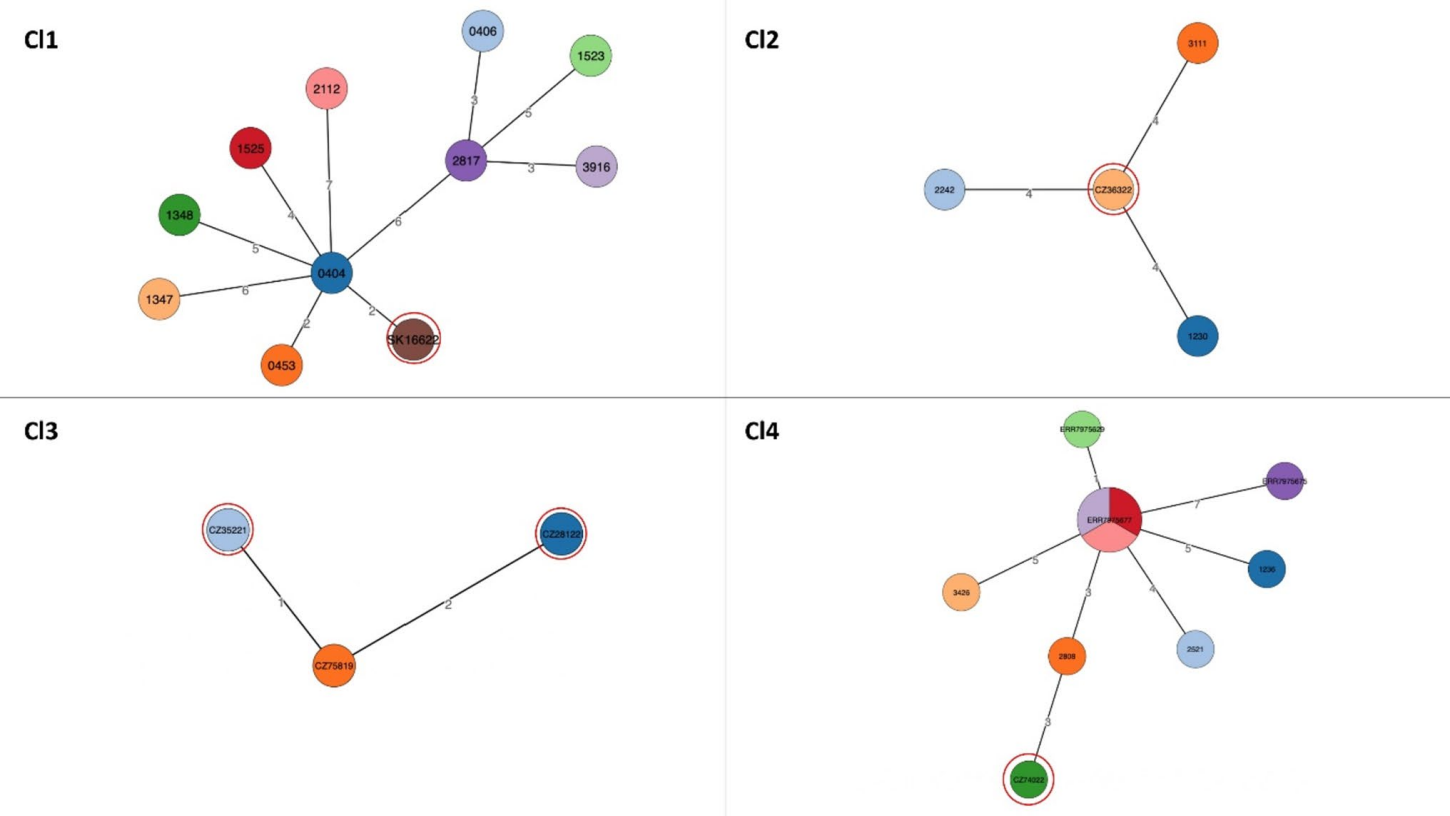
Minimum spanning trees based on SNP differences between the strains, including those isolated from Ukrainian patients in the Czech Republic and Slovakia. The red outline highlights the patient classified as a Ukrainian war refugee or migrant. Cl—cluster, CZ—Czech Republic, SK—Slovakia; other clustered patients in Cl1, Cl2 and, Cl4 were notified in Ukraine

We explored possible international connections and obtained a more in-depth understanding of the source of TB disease among certain war refugees diagnosed in the host countries. The two largest clusters (Fig. 5) were detected, comprising the sensitive (Cl1) and MDR (Cl4) *MTBC* isolates (Beijing sublineage) from Ukrainian refugees and isolates from previous studies conducted in Ukraine. This indicates a national chain of transmission spanning over 14 years, as clustered isolates were collected in microbiological laboratories throughout Ukraine during the countrywide study from 2009 to 2014 and in a prison hospital in Kharkiv from 2018 to 2019. The presented results confirmed the theory that most cases resulted from local transmission in Ukraine (without known epidemiological links).

One other cluster (Cl3; Fig. 5) comprised three Ukrainian migrants diagnosed in 2019, 2021 and 2022. Retrospective contact tracing confirmed that these patients had an epidemiological link, mainly between household contacts. The presented results confirmed the theory that most cases resulted from local transmission in Ukraine (without known epidemiological links).

The limited clustering observed between strains from recent migrants/refugees and patients from the host countries indicated their lack of relatedness, thus suggesting that transmission is improbable within the Czech Republic and Slovakia.

Reported transmission events confirmed an increased risk of transmission in lineage 2 compared to other lineages (*p* < 0.0001; OR: 19.5; 95% CI 4.073–93.03).

## Discussion

TB in war refugees/migrants represents an important clinical and public health threat, particularly in low TB-incidence countries. In this two-country study, we found that the clustering rate between strains from recent Ukrainian war refugees/migrant is low, and therefore transmission is unlikely to have occurred in host countries, and these patients are part of larger clusters previously identified in Ukraine.

In recent decades, Ukrainians represented a substantial proportion of foreign-born patients diagnosed with TB in the Czech Republic and Slovakia, and the incidence of TB among Ukrainians has shown a declining trend. Nevertheless, the onset of war and large migration brought alarming public health concerns regarding TB [19]. The recently published data showed the increased incidence of TB in low-incidence countries correlating with an increasing prevalence of TB among migrants originating from countries with a high TB burden [20]. Consequently, we anticipate more TB patients in the Czech Republic and Slovakia, considering the typical incubation period of TB disease, which spans from several months to 2 years. The incidence curve in the Czech Republic experienced a sharp decline starting in 2003 and has remained relatively stable since then. Similarly, the incidence of TB in Slovakia has decreased significantly since 2013, with a slight increase in incidence in 2022. As expected, the data from 2022 demonstrate historically the highest number of Ukrainian patients with TB in the Czech Republic (incidence 13.99 per 100,000 population) and Slovakia (incidence 7.65 per 100.000 population). The TB case numbers among individuals arriving from Ukraine appeared to be lower than initially anticipated in several other countries with a low incidence of TB [21, 22]. The relatively low number of TB patients among war refugees in Slovakia can be attributed to the rather significant mobility of this population, which did not necessarily stay in the country for an extended period. However, there is a lack of data in the country to confirm this hypothesis. Almost 23% of *MTBC* isolates were classified as RR/MDR. These results are consistent with the estimated proportion of RR/MDR-TB in Ukraine in 2021 [8]. However, it is important to note that the recorded number of cases vs estimated case numbers is lower, probably due to delayed or inadequate diagnosis of TB and reporting delays, as universal testing for TB infection, as well as screening for active TB among refugees arriving in European countries from Ukraine is not recommended by ECDC [23]. Recent results highlight the utility of an active screening strategy (including systematic full health consultation and a chest X-ray) in people coming from Ukraine to estimate a more accurate prevalence of TB and primary MDR form among the target population [22]. For example, recent statistics from the WHO suggest that approximately 230 Ukrainian refugees in Poland are estimated to have drug-resistant tuberculosis, yet only 46 individuals are presently undergoing treatment [24].

As expected, our findings revealed that the genetic diversity of *MTBC* isolates among Ukrainian war refugees and migrants is mainly driven by sublineages 2.2.1 (24/91), 4.1.2.1 (20/91) and 4.8 (19/91). Previous studies conducted in Ukraine, the Russian Federation and other Central Asian countries showed similar results [25–27]. The results also showed changes in the distribution of lineages in Slovakia, with a predominance of the Beijing lineage, which occurred in only 2 patients over the last 6 years [16]. As this lineage is characterized by increased virulence and transmissibility, we strongly recommend public health authorities to take the necessary measures, such as a thorough investigation of close contacts, as early detection facilitates rapid initiation of an appropriate treatment regimen [28].

Similar to other studies, cluster analysis revealed that most infections were likely the result of reactivation of latent disease or exposure to TB before migration rather than recent transmission occurring within the host country [29, 30]. The largest clusters comprised war refugees diagnosed in the Czech Republic, TB patients from various regions of Ukraine, and incarcerated individuals diagnosed with pulmonary TB at a specialized facility in the Kharkiv region, Ukraine.

One limitation of our study was the inability to obtain social–demographic information from the participants we enrolled. As a result, we could not ascertain the epidemiological associations among the clustered individuals. Additionally, the sample collection didn't cover all Ukrainian war refugees and migrants with TB in the Czech Republic and Slovakia during the study period, as some patients had treatment plans before arrival. Also, the low clustering rate was influenced by limited sequencing data availability from Ukraine.

Our study is the first comprehensive molecular–epidemiology investigation on the impact of Ukrainian war migration on TB transmission in low-incidence countries. Although we did not observe a significant increase in the incidence of TB among the population in host countries, we anticipate that the effects of migration could become evident in the forthcoming years. The large host population dilutes the higher incidence among refugees, unless a major outbreak occurs. Finally, our results highlight the ability of WGS to perform genomic investigations across time and geography and underscore the urgent need to enhance and reinforce current initiatives aimed at the early detection and treatment of TB and to increase awareness and understanding of the hazards associated with huge migration events from high-incidence TB countries.

### Supplementary Information

Below is the link to the electronic supplementary material.

Supplementary file 1 (DOCX 361 kb)

## Data Availability

Sequence data relating to all isolates have been deposited in the Sequence Read Archive under BioProject numbers—PRJNA994428: isolates from Ukrainian war refugees and migrants; PRJNA994428 (sensitive strains) and PRJNA886608 (MDR, pre-XDR strains): isolates from TB patients diagnosed in Slovakia; PRJEB48710 (mono- and polyresistant strains) and PRJNA886608 (MDR, pre-XDR strains): isolates from TB patients diagnosed in Czech Republic; PRJNA994428 (strains from prison inmates) and SRP128089: isolates from TB patients diagnosed in Ukraine.

## Abbreviations

TB: Tuberculosis
WGS: Whole genome sequencing
MTBC: Mycobacterium tuberculosis
MDR: Multi-drug-resistant
RR: Rifampicin-resistant
TST: Tuberculin skin test
WHO: World Health Organization
ECDC: European Centre for Disease Prevention and Control
pDST: Phenotypic drug susceptibility testing
LJ: Lowenstein–Jensen
PZA: Pyrazinamide
CC: Critical concentrations
