# Correction: Tuberculosis in Ukrainian War Refugees and Migrants in the Czech Republic and Slovakia: A Molecular Epidemiological Study

**DOI:** 10.1007/s44197-024-00212-w

**Published:** 2024-02-26

**Authors:** Matúš Dohál, Věra Dvořáková, Miluše Šperková, Arash Ghodousi, Maryam Omrani, Igor Porvazník, Erik M. Rasmussen, Mária Škereňová, Michaela Krivošová, Olha Konstantynovska, Timothy M. Walker, Vladyslav Nikolayevskyy, Daniela M. Cirillo, Ivan Solovič, Juraj Mokrý

**Affiliations:** 1 Biomedical Centre Martin, Jessenius Faculty of Medicine in Martin, Comenius University Bratislava, Malá Hora 4A, 036 01 Martin, Slovak Republic; 2 National Reference Laboratory for Mycobacteria, National Institute of Public Health, Prague, Czech Republic; 3 Emerging Bacterial Pathogens Unit, IRCCS San Raffaele Scientific Institute, Milan, Italy; 4 Vita-Salute San Raffaele University, Milan, Italy; 5 Center for Multidisciplinary Research in Health Science-MACH, University of Milan, Milan, Italy; 6 National Institute of Tuberculosis, Lung Diseases and Thoracic Surgery, Vyšné Hágy, Slovakia; 7 Faculty of Health, Catholic University, Ružomberok, Slovakia; 8 International Reference Laboratory of Mycobacteriology, Statens Serum Institut, Copenhagen, Denmark; 9 Department of Clinical Biochemistry, Jessenius Faculty of Medicine in Martin, Comenius University, Bratislava, Martin, Slovakia; 10 V.N. Karazin Kharkiv National University, Kharkiv, Ukraine; 11 Oxford University Clinical Research Unit, Ho Chi Minh City, Vietnam; 12 Nuffield Department of Medicine, Centre for Tropical Medicine and Global Health, University of Oxford, Oxford, UK; 13 Department of Infectious Diseases, Faculty of Medicine, Imperial College London, London, UK; 14 Department of Pharmacology, Jessenius Faculty of Medicine in Martin, Comenius University Bratislava, Martin, Slovak Republic

**Correction: Journal of Epidemiology and Global Health** 10.1007/s44197-023-00166-5

After the publication of the article, the author asked to augment the affiliations of the author group. The affiliations should read like this—the changes are given bold):

^1^ **Biomedical Centre Martin, Jessenius Faculty of Medicine in Martin**, Comenius University Bratislava, Martin, Slovak Republic

^2^ **National Reference Laboratory for Mycobacteria**, National Institute of Public Health, Praha, Czech Republic

^3^ **Emerging Bacterial Pathogens Unit**, IRCCS San Raffaele Scientific Institute, Milan, Italy

^4^ **Vita-Salute** San Raffaele University, Milan, Italy

^5^ **Center for Multidisciplinary Research in Health Science-MACH**, University of Milan, Milan, Italy

^6^ National Institute of Tuberculosis, Lung Diseases and Thoracic Surgery, Vyšné Hágy, Slovakia

^7^ **Faculty of Health**, Catholic University, Ružomberok, Slovakia

^8^ **International Reference Laboratory of Mycobacteriology**, Statens Serum Institut, Copenhagen, Denmark

^9^ **Department of Clinical Biochemistry, Jessenius Faculty of Medicine in Martin, Comenius University, Bratislava, Martin, Slovakia**

^10^ V.N. Karazin Kharkiv National University, Kharkiv, Ukraine

^11^ Oxford University Clinical Research Unit, Ho Chi Minh City, Vietnam

^12^ **Centre for Tropical Medicine and Global Health, Nuffield Department of Medicine**, University of Oxford, Oxford, UK

^13^ **Department of Infectious Diseases, Faculty of Medicine**, Imperial College London, UK

^14^ **Department of Pharmacology, Jessenius Faculty of Medicine in Martin**, Comenius University Bratislava, Martin, Slovak Republic

*e-mail: matus.dohal@uniba.sk, address: Malá Hora 4A, 036 01, Martin, Slovakia

The Original article has been updated.

