# Supplementary material for: Tuberculosis in Ukrainian War Refugees and Migrants in the Czech Republic and Slovakia: A Molecular Epidemiological Study
Source: J Epidemiol Glob Health. 2023 Dec 4;14(1):35–44. doi: 10.1007/s44197-023-00166-5 (PMC11043285; doi:10.1007/s44197-023-00166-5)
Supplement: Supplementary file 1 — (DOCX 361 kb) [file 44197_2023_166_MOESM1_ESM.docx]

**Supplementary Data**

1. **DNA extraction from isolates collected in Ukraine**
   1. **Isolates collected between November 2009 and May 2014**

To extract and purify high-quality genomic DNA for next-generation sequencing, commercially available (para)magnetic- (Maxwell 16 Cell DNA Purification kit, Promega, Madison, WI, USA; NucliSENS easyMag, Biomerieux, Marcy l'Etoile, France), and column-based (QIAamp DNA Mini Kit, Qiagen, Hilden, Germany) systems, or CTAB (N-cetyl-N,N,N-trimethyl ammonium bromide)/NaCl protocol were used according to manufacturer’s instructions and published procedures.

- 1. **Isolates from prison inmates obtained from V.N. Karazin Kharkiv National University, Kharkiv, Ukraine**

These were sequenced using the following protocol: genomic DNA was extracted using 1 mL of MGIT culture, a lysis buffer (NaCl, Triton, EDTA, TRIS), and protease K. The heat inactivation step was performed at 95°C/30 minutes. Total DNA was purified using the Promega Maxwell 16-cell DNA purification kit (AS1020), and the final concentration was quantified by a Qubithigh sensitivity DNA assay (Life Technologies) spectro-photometer with a fluorescent detection system (Qubit 3.0 Fluorometer). A library preparation was performed by the Nextera XT DNA Library prep kit (Illumina, San Diego). All isolates were sequenced on the Illumina NextSeq500 System (Illumina) according to the manufacturer’s instructions.


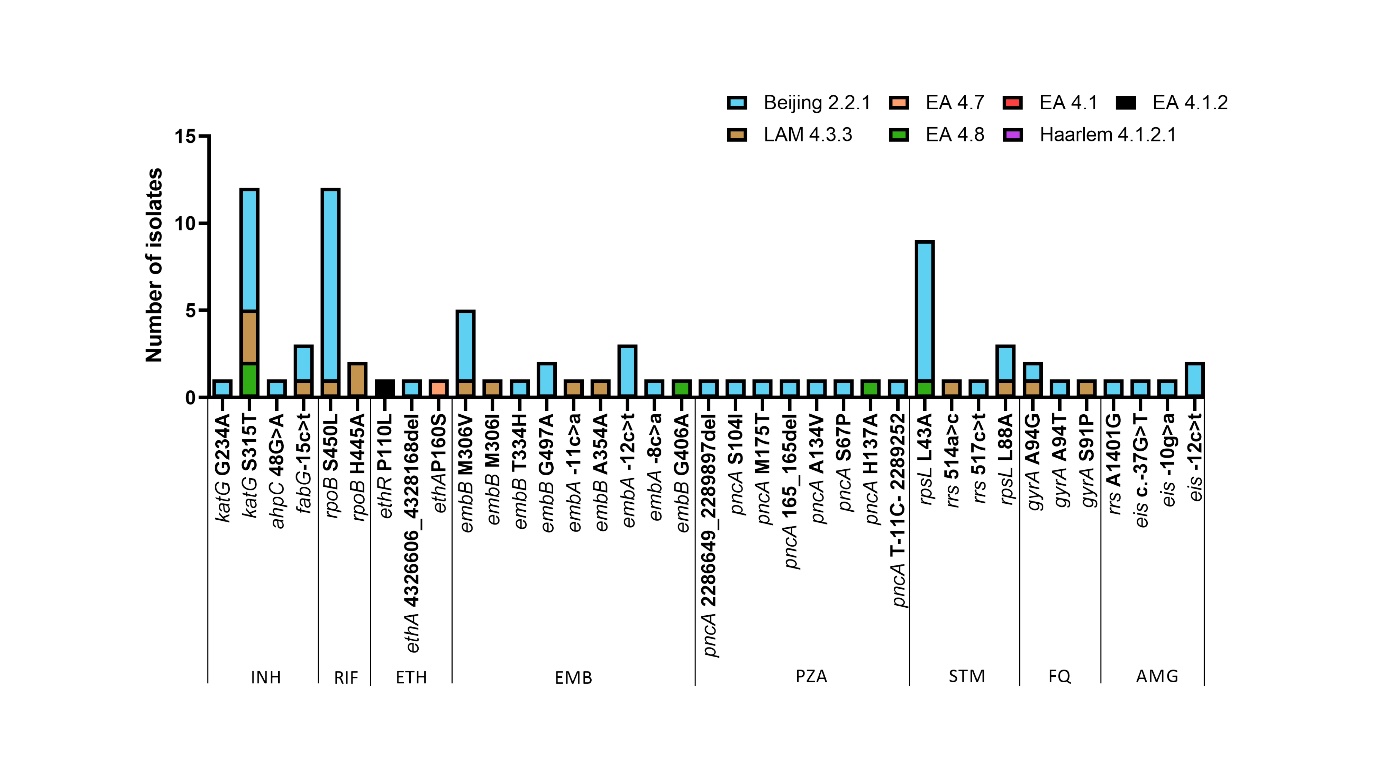
**
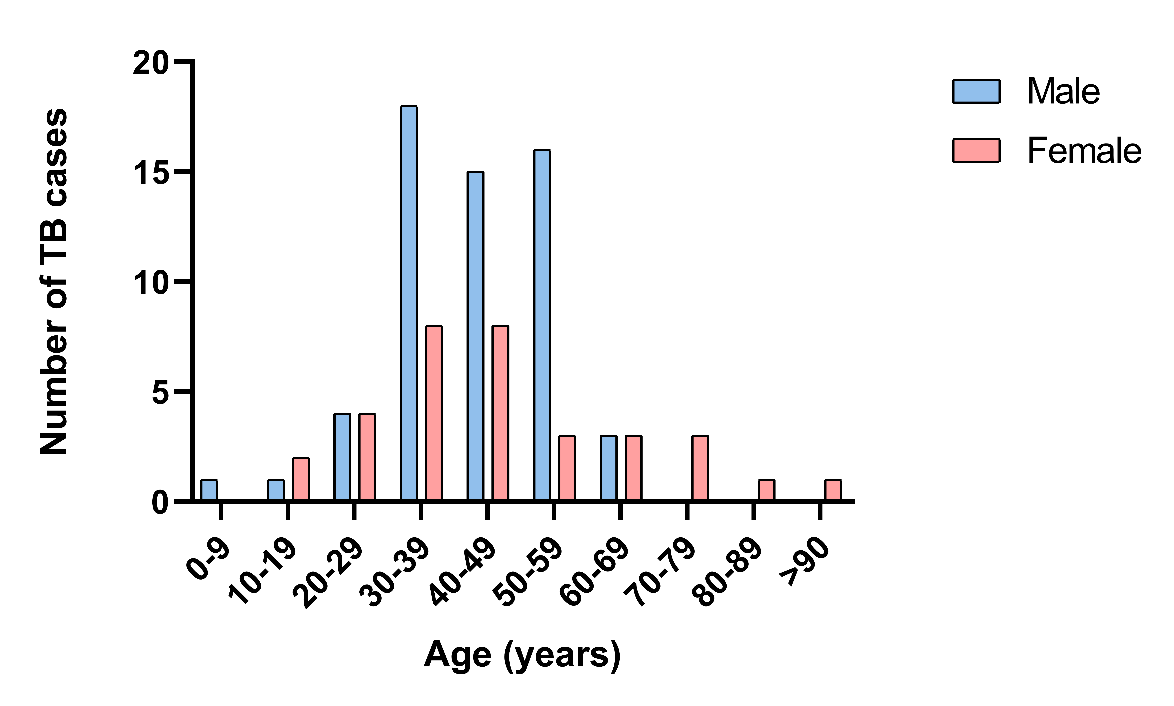
Supplementary Figure 1.** Number of reported TB cases in Ukrainian refugees and migrants, by age group and sex, in the Czech Republic and Slovakia.

**Supplementary Figure 2**. Correlation of genotypic drug resistance and phylogenic lineages of Mtb isolates among Ukrainian refugees and migrants. INH, isoniazid; RIF, rifampicin; EMB, ethambutol; ETH, ethionamide; PZA, pyrazinamide; STM, streptomycin; FQ, fluoroquinolones; AMG, aminoglycosides; PAS, para-aminosalicylic acid; EA, Euro-American; LAM, Latin American Mediterranean


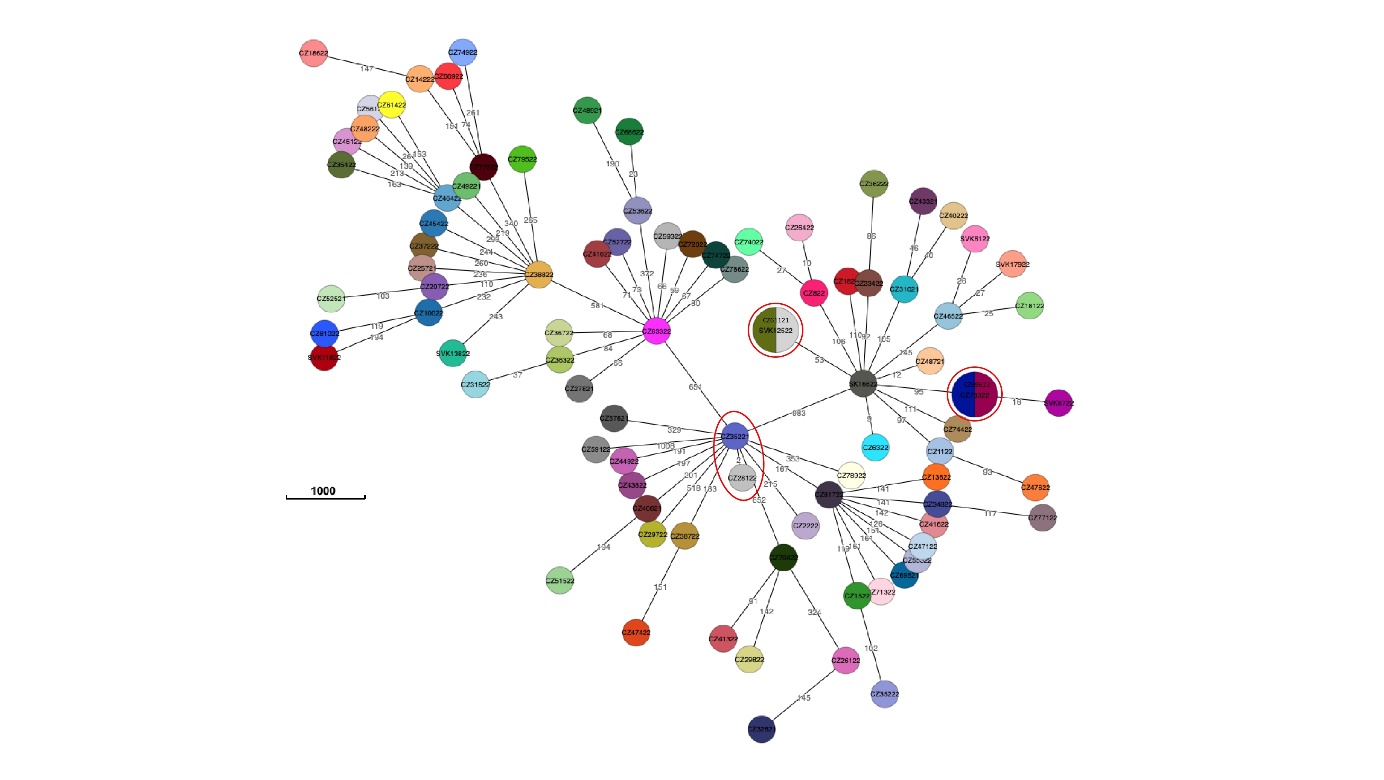
**Supplementary Figure 3.** Minimum spanning tree based on a maximum of 5 SNPs difference between the 91 strains isolated from Ukrainian war refugees and migrants during the study period
